# Case Report: Cavernous hemangioma of rib: an extremely rare venous malformation but easily misdiagnosed as aggressive tumors

**DOI:** 10.3389/fonc.2023.1164331

**Published:** 2023-06-07

**Authors:** Shan Liu, Luan Xiang, Fan Ding, Ling Yuan, Xiu-Mei Wang

**Affiliations:** ^1^Department of Thoracic Surgery, The Third Hospital of Wuhan, Wuhan, China; ^2^Department of Thoracic Cardiovascular Surgery, General Hospital of Central Theater Command of the People’s Liberation Army, Wuhan, China; ^3^Department of Orthopaedics, General Hospital of Central Theater Command of The People’s Liberation Army, Wuhan, China; ^4^Department of Pathology, General Hospital of Central Theater Command of the People’s Liberation Army, Wuhan, China

**Keywords:** hemangioma, rib, computerized tomography, malignancy tumor, case report, venous bony malformation

## Abstract

Hemangioma is a congenital vascular malformation that occurs commonly in the skin and soft tissues of younger individuals but rarely in the bone. The term hemangioma occurring in bone has been referred to as venous bony malformation also. In these rare cases, vertebral bodies occur more often, followed by the craniofacial skeleton and long bones. Most rib tumors are malignant, and hemangiomas of the rib tend to grow expansively and disrupt the cortex. Venous malformations in ribs are not tumors but can be misdiagnosed as aggressive tumors or infectious processes. In fact, hemangioma of bone is a locally aggressive benign vascular malformation associated with a good prognosis. To date, no more than 50 cases of rib hemangiomas have been described in the English literature. This report presents a case of an asymptomatic 27-year-old female patient who found a quail egg-sized lump on the right side of her chest that was the size of a cocoon 2 months prior. Then, the lump grew rapidly to the size of an egg when she presented to the hospital. Because of its worrisome histomorphologic features and aggressive clinical radiologic findings, it was once misdiagnosed as a malignant tumor by most doctors. However, the pathological results after the operation confirmed rib hemangioma. Therefore, this case report aims to share this particular case so that more doctors can better understand the particularity of this disease’s progression.

## Introduction

Hemangioma is a benign malformation of blood vessels that can occur throughout the body (1, 2). The term hemangioma occurring in bone has been referred to as venous bony malformation also (3, 4). Bone hemangiomas comprise disorganized channels of abnormal blood vessels lined with endothelialized sinusoidal channels, predominantly of the cavernous hemangioma type (5). We reported this case of cavernous hemangioma due to its extremely rare occurrence in the 10th rib. We obtained a variety of diagnostic data on this patient, such as chest computed tomography (CT), magnetic resonance imaging, bone nuclear medicine imaging, and postoperative pathology. We hope for this case report of an asymptomatic patient to be considered part of the differential diagnosis of rib tumors.

## Case report

**Figure 1:**
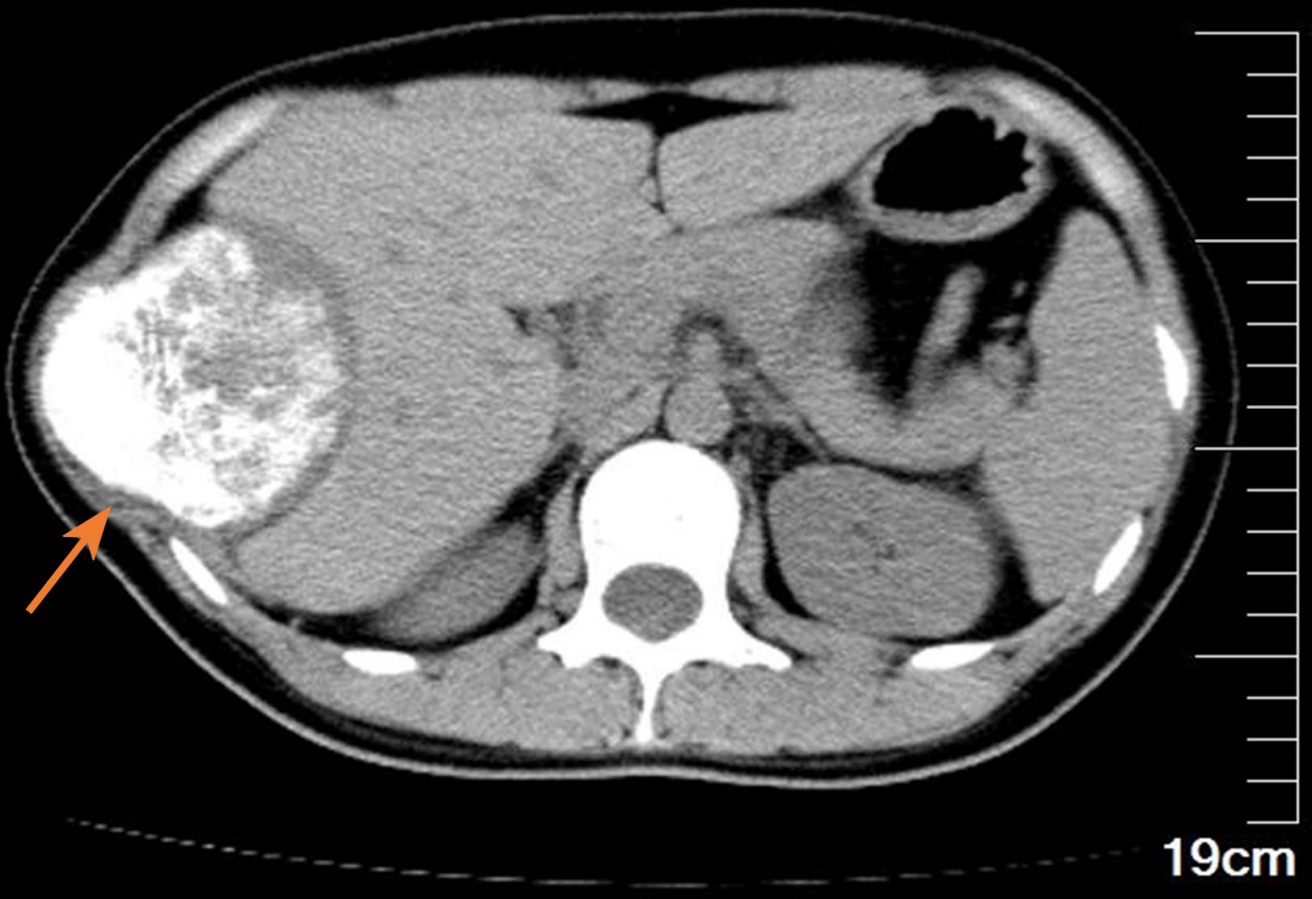
The chest CT mediastinum window showed a huge lump (see the arrow) compressing the liver, the lump’s internal density was uneven, with extensive calcification-like high-density shadows, and the 10th rib on the right was invaded and destroyed by the lump.

**Figure 2:**
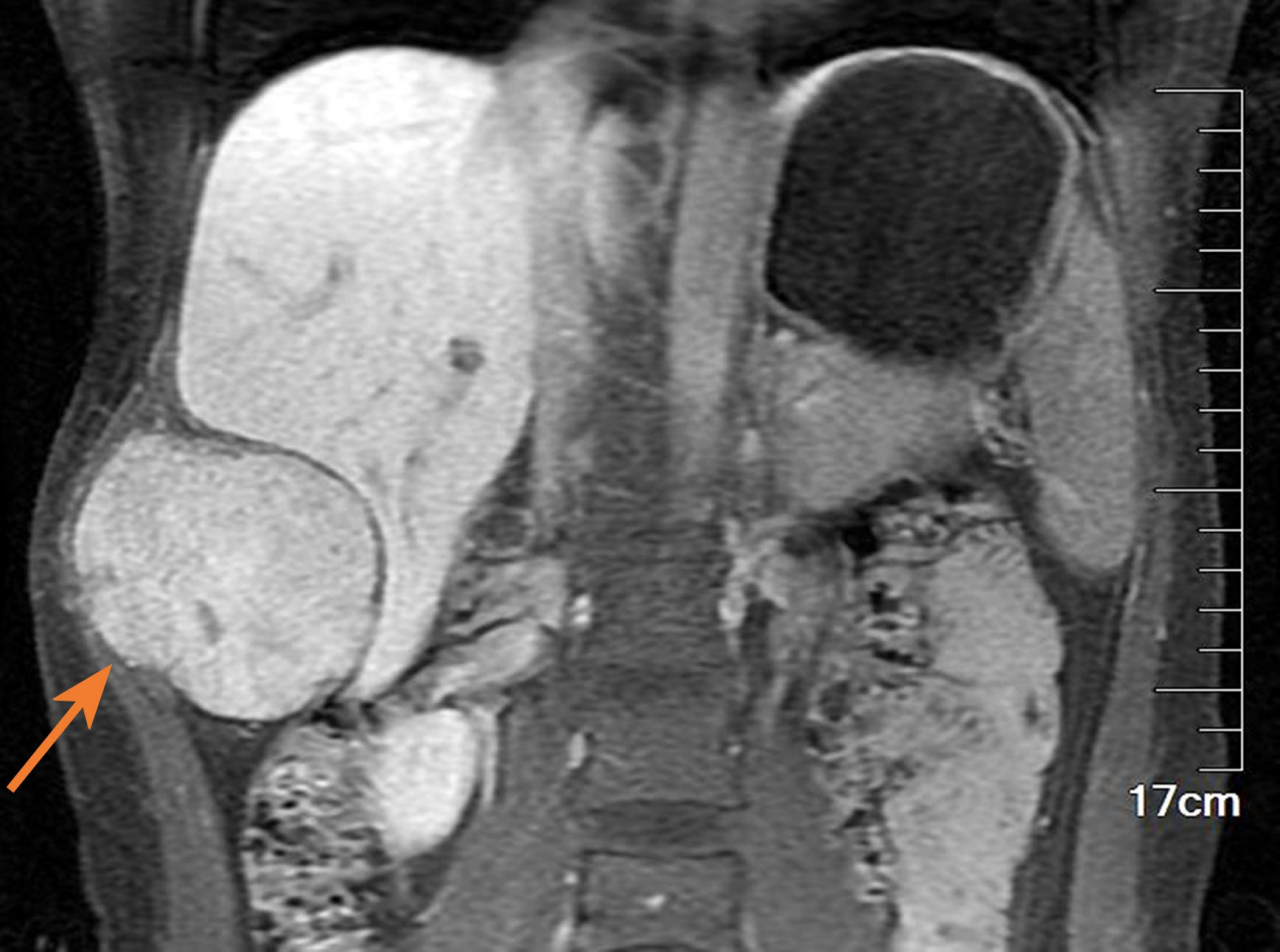
The lump (see the arrow) presented a high signal with a clear boundary between the lump and the lung, but the lump margin was rough and characterized by destruction (MRI T2-weighted images).

**Figure 3:**
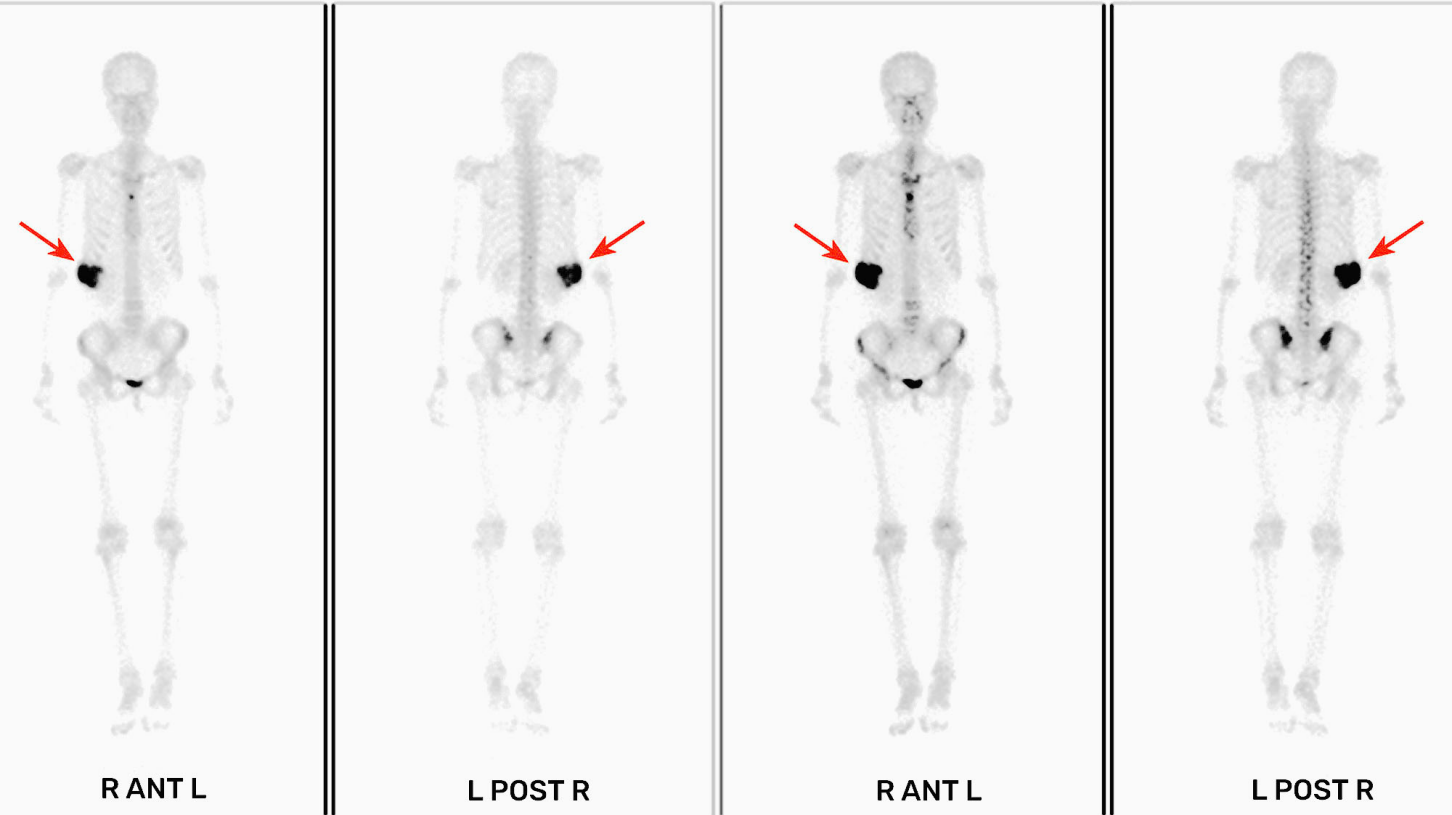
The right chest wall presented a shadow (see the arrow) of abnormal radiation accumulation (whole-body bone imaging 3 h after intravenous injection of ^99m^Tc-MDP).

**Figure 4:**
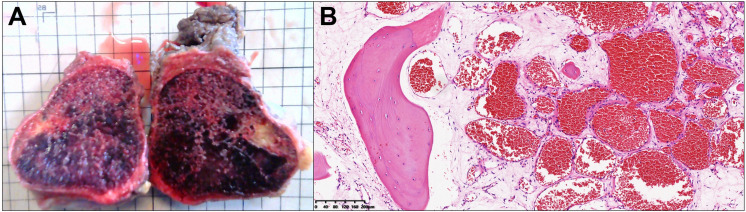
The lump presents an irregular spherical shape. The inside appears as grid-like and honeycomb-like vascular tissue after incision **(A)**; each grid is 1 × 1 cm in size). The tissue slices show enlarged blood vessels, a large number of red blood cells in the blood vessels, and transparent fibrous matrix around the blood vessels **(B)**; hematoxylin and eosin staining original, ×40).

A 27-year-old female patient was admitted to the hospital after finding a lump in the right chest wall for more than 2 months. Approximately 2 months ago, a quail egg-sized lump in the right chest wall attracted the patient’s attention. However, the patient did not feel any discomfort, such as pain or numbness. Therefore, the patient initially disregarded the lump. However, in the following 2 months, the lump grew rapidly, which prompted the patient to present to the hospital. Recently, she had no fever or other discomfort and no local skin redness or swelling. Physical examination showed that an irregular round lump the size of an egg was palpable at the intersection of the right mid-axillary line and the 10th rib. The skin on the surface of the lump had no abnormal changes, the lump was hard in texture, the boundary between the lump and surrounding tissues was blurred, and the lump could not be moved, but its surface felt smooth. There was no pain or sensitivity when touching the lump area. All serological examination results were normal, and there was no evidence of inflammation. Chest CT examination showed a large lump in the right chest wall, approximately 7.3 × 8.0 × 6.8 cm in size. The internal density of the lump was uneven, showing chaotic and complex high-density shadows, with string-like high-density ossification changes. The edge of the lump was smooth, without burrs, and did not invade surrounding tissues. The distal end of the right 10th rib was obviously expansive and damaged. The lump compressed the liver, but the boundary between the lump and the liver was clear (see **Figure 1**). The possibility of chondrosarcoma was considered by CT results. Chest diffusion-weighted imaging (DWI) showed that the lump grew on the 10th rib and presented uneven enhancement (DWI images high signal), and the liver was significantly compressed, but no abnormal signal was observed in the liver (see **Figure 2**). The result of chest DWI was suggestive of chondrosarcoma. Whole-body bone imaging revealed an abnormal circular concentration shadow of radiation on the right chest wall, and the radiation uptake ability was significantly increased (see **Figure 3**). The patient was prepared to undergo right chest wall tumor resection. The pathological results of intraoperative rapid freezing showed cavernous hemangioma of the rib, and all incision margins were negative. A firm, well-circumscribed mass with dimensions of 8.2 cm × 8.0 cm × 7.1 cm was found arising from the medullary cavity of the 10th rib (**Figure 4A**). On the cut surface of the lump, it had a multicapsular appearance. It bulged out from the inner surface of the 10th rib toward the thoracic cavity. There was a significant disruption of the adjacent 10th rib bone, as well as the presence of a hyperplastic vasoganglion. According to a microscopic examination of the lump, it consisted of irregularly distorted blood vessels without outward signs of necrosis or pleomorphism (**Figure 4B**). The final pathologic diagnosis was cavernous hemangioma of the rib. The postoperative course was uneventful, and the patient was discharged 5 days after surgery. We have followed up the patient regularly for approximately 3 years. CT or X-ray examinations were performed at each follow-up. We found no evidence of lump recurrence and no symptoms of discomfort.

## Discussion

Bone hemangiomas are benign malformations that have small or large vascular channels (6, 7). Typically, hemangiomas of bone involve vertebral bodies, followed by the craniofacial skeleton and long bones (6). Cavernous hemangioma of the rib is an extremely rare benign malformation involving the bone marrow and cortex to help (8, 9). To date, no more than 50 cases of hemangioma of the rib have been described in the English literature in PubMed. We considered that cavernous hemangioma of the rib has the following characteristics by studying and analyzing all the previously reported cases (2–37). Previous literature has found that the incidence of this disease mainly has the following clinical characteristics. First, this disease has been reported relatively frequently in East Asia, with most cases originating in China and Japan and occasionally in other countries (2–37). Second, there was no significant difference in the incidence of men and women from a gender perspective (2–37). Third, most patients were older than 45 years (range, 11–76 years) (2–37). Fourth, although malformations were often large (ranging from 3 to 16 cm in size; median, 6 cm), most patients did not have typical clinical symptoms, and malformations were typically detected incidentally (2–37). Fifth, the sixth to ninth ribs were areas of high incidence, and there was no difference in the left or right sides (2–37). Sixth, cavernous hemangioma of the rib usually only occurs in one rib, and the lump grows to the thoracic cavity and chest wall at the same time, which causes compression on the surrounding organs or tissues and may promote corresponding symptoms (2–37).

Although rib hemangiomas are predominantly incidental findings that are asymptomatic, there have been reports of symptomatic presentations such as chest pain, swelling, shortness of breath, intercostal paresthesia, backaches with pleural effusion, and thoracic outlet syndrome (29). Previous reports showed that the malformations did not grow rapidly; in our case, the malformation only took less than 2 months to grow into a large chest wall lump, which might be related to the age of the patient (23).

Chest X-ray examination manifested as a strip or round abnormal shadow in a rib of the chest, which may be accompanied by bone destruction of the rib. It presents as a round and irregular lump on chest CT, with uneven-density and high-density shadow lump-like calcification and rib cortical disruption (2–37). MRI is an important adjunct; T1-weighted images usually show the reticular architecture of the medullary component and the fatty content, and T2-weighted images usually show hyperintense lesions due to an abundance of large vascular channels (29). There are, however, certain imaging findings that are also seen in tumors that are malignant, such as chondrosarcomas or Ewing sarcomas. There may not be useful information available from PET/CT to differentiate a malignant tumor from a rib hemangioma due to the rib hemangiomas’ higher ^18^FDG uptake (30).

As there is a lack of literature available to guide, we remain unable to recommend how to best treat rib hemangiomas. All but one previous case report has been treated with resection to definitively rule out cancer. Although this case of non-operative management of rib hemangioma with a 1-year follow-up did not reveal any malignant degeneration or size increase, rib hemangiomas required surgical intervention sooner or later, as they could not heal spontaneously (29, 30). Moreover, the malformations contain rich vascular channels, and a biopsy may cause bleeding. Due to malformation seeding risks through the biopsy tract, some authors recommend avoiding biopsy (6). In addition, considering that venous malformations of rib malformations are often malignant and rib hemangiomas often have imaging findings of cortical disruption, we advocate aggressive surgical resection. For rib hemangiomas, the widely accepted surgical approach is to remove the primary rib along with the malformations (2–37). To achieve optimal oncological outcomes, an excision margin of at least 2 mm is recommended because this margin is considered a safe margin for high-grade chondrosarcomas according to a previous report (6). Thoracic reconstruction should be required if the lump is large in size. No further treatment is necessary after the operation, but regular examinations are still needed.

## Conclusions

In conclusion, the clinical manifestations and imaging examination results of cavernous hemangioma of the rib showed characteristics of malignant bone tumors, such as osteosarcoma and chondrosarcoma. Cavernous hemangioma of the rib should be considered for chest wall tumors conforming to the above characteristics. Surgical resection is still the key measure to manage cavernous hemangioma of the rib. We maintain a cautious attitude toward adopting needle biopsy to clarify the histopathological results of chest wall lumps with the above clinical characteristics before surgery. In addition, we hope that this particular case could be shared to provide materials for clinical teaching or peer reference or further research.

## Data availability statement

The original contributions presented in the study are included in the article. Further inquiries can be directed to the corresponding authors.

## Ethics statement

Ethical review and approval were not required for the study on human participants in accordance with the local legislation and institutional requirements. The patient provided written informed consent to participate in this study. Written informed consent was obtained from the individual for the publication of any potentially identifiable images or data included in this article.

## Author contributions

SL, LX and FD wrote the manuscript, collected the data, and analyzed the data. X-MW and LY designed this study and checked and provided guidance during the manuscript writing process. All the authors participated in the diagnosis, treatment, and follow-up of the patient and participated in the collection and arrangement of medical records. All authors have agreed to submit the manuscript in its current form and to revise and publish it according to the journal review comments. All authors have agreed to select the current journal for submission and have agreed to be accountable for all aspects of the work.
